# UVB Irradiation Regulates ERK1/2- and p53-Dependent Thrombomodulin Expression in Human Keratinocytes

**DOI:** 10.1371/journal.pone.0067632

**Published:** 2013-07-02

**Authors:** Huey-Chun Huang, Tsong-Min Chang, Yu-Jia Chang, Hsin-Yun Wen

**Affiliations:** 1 Department of Medical Laboratory Science and Biotechnology, College of Health Care, China Medical University, Taichung, Taiwan; 2 Department of Applied Cosmetology, Hung Kuang University, Taichung, Taiwan; 3 Department of Surgery, Taipei Medical University, Taipei, Taiwan; University of Tennessee, United States of America

## Abstract

Thrombomodulin (TM) is highly expressed in endothelial cells and acts as a natural anticoagulation factor to maintain circulation homeostasis. TM is an interesting molecule with many physiological functions, including anti-inflammation, anti-thrombosis, and carcinogenesis inhibition. TM can also be detected on the spinous layer of epidermal keratinocytes. However, the role of epidermal TM is still under investigation. In this study, we investigated keratinocyte TM expression and regulation in response to sub-cytotoxic ultraviolet B (UVB) irradiation. Oxidative stress was assessed with DCF and the results revealed that UVB irradiation significantly and dose-dependently augmented reactive oxygen species (ROS) production in HaCaT cells. In addition, low-dose UVB irradiation decreased TM mRNA and protein levels. Blocking ROS production and ERK activation prevented UVB-induced TM down-regulation. The nuclear p53 accumulation and TM promoter binding was observed within 3 h after UVB exposure. Small interfering RNA-mediated p53 knockdown disrupted the UVB-mediated TM protein down-regulation. Our study demonstrates that UVB irradiation results in ROS accumulation and ERK activation, which causes the nuclear p53 accumulation and TM promoter binding to inhibit TM expression. This study provides novel evidence demonstrating that p53 serves as a key regulator of keratinocyte TM expression.

## Introduction

In normal human skin, thrombomodulin (TM) is expressed in the keratinocyte suprabasal spinous layers where keratinocytes start to differentiate and make extensive contacts with surrounding keratinocytes [1]. Previous reports suggest that skin TM may regulate keratinocyte differentiation and modulate fibroblast collagen production during cutaneous wound healing [2], [3]. TM demonstrates anti-inflammatory properties on UV irradiation-induced cutaneous inflammation [4]. Conversely, burned areas of the skin that exhibited negative TM staining caused pro-inflammatory cytokine release [5]. In addition, clinical studies also revealed an inverse correlation between TM expression and skin disease progression [6]–[8]. Therefore, TM is functionally active and plays crucial homeostatic roles in skin epidermis.

Ultraviolet (UV) irradiation from sunlight is the major environmental cause of skin cancer. Previous reports have demonstrated that exposure of skin to UVB causes several detrimental skin effects including inflammation, immuno-suppression [9], premature skin aging, and skin cancer development [10], [11]. At the molecular level, UVB can also induce intracellular reactive oxygen species (ROS) generation [12], [13], which activates cell signaling and stimulates transcription factor expression such as nuclear factor kappa B (NF-κB), activator protein-1 (AP-1) and p53 [14]–[17]. These transcription factors play important roles in photo-carcinogenesis and photo-aging [14], [15].

Several factors down-regulate TM expression, which contributes to inflammation-associated [18]. Conversely, TM expression up-regulation may be a protective mechanism to compensate for pro-inflammatory effects [19]. Natural anti-inflammatory pathway dysfunction may be particularly problematic because TM dampens inflammatory responses [20]. TM expression in endothelial cells is suppressed by pro-inflammatory cytokines such as TNF-α [21]. The pro-inflammatory cytokine-mediated TM inhibition was mediated by NF-κB [22]. Because UVB exerts inflammation, we postulate that UVB may exhibit inhibitory effects on keratinocyte TM expression. In the present study, both human primary epidermal keratinocytes, as well as HaCaT cells, were used to elucidate the regulatory mechanism of UVB on TM expression, and the role of p53 in regulation of keratinocyte TM expression was also investigated.

## Materials and Methods

### Cell Culture and UVB Irradiation

HaCaT cells [23] were maintained in DMEM (Hyclone, Logan, UT) supplemented with 10% fetal bovine serum and 1% antibiotics at standard cell culture conditions (37°C, 5% CO_2_ in a humidified incubator). Human epidermal keratinocytes (Cell Application, San Diego, CA) were cultured in keratinocyte growth media (Cell Application, San Diego, CA) supplemented with 10% fetal bovine serum and 1% antibiotics. UVB was generated from a 15 W UVB lamp equipped with an electronic controller at a distance of 30 cm. The UVB dose was calculated accurately with a UVB meter (UVP, Upland, CA). Irradiation doses were calculated using the formula: dose (mJ/cm^2^) = exposure time (sec.)×intensity (mW/cm^2^) [24]. For UVB irradiation, HaCaT cells were kept in phosphate buffered saline (PBS) and irradiated with 3 mJ/cm^2^ UVB (wavelength 290–320 nm). After UV irradiation, fresh media was added to each plate, and cells were maintained in regular culture conditions for a designated time until analysis.

### Trypan Blue Exclusion Assay

A trypan blue exclusion assay was used to assess the effect of UVB on HaCaT cell growth and viability. Briefly, following treatment with UVB, cells were trypsinized and pelleted by centrifugation, and the cell pellet was re-suspended in 300 µL DMEM media. Trypan blue (0.4% in PBS, 10 µL) was added to a smaller aliquot (20 µL) of cell suspension, and the cell number (viable unstained and nonviable blue) was counted using a hemocytometer under the microscope. Each sample was counted in duplicate, and each experiment was repeated at least three times.

### TM Activity Assay

HaCaT cells were split into a 96-well plate at a density of 2×10^4^ cells/well and were allowed to attach overnight. The cells were washed in a buffer containing 20 mM Tris (pH 7.4), 0.15 M NaCl, 2.5 mM CaCl_2_, and 5 mg/mL bovine serum albumin (BSA) and were incubated with 40 µL reaction mixture (37.5 nM thrombin and 5 µg/mL protein C in wash buffer) at 37°C for 30 min. Protein C activation was terminated by adding 40 µL antithrombin III (6 IU/mL) and heparin (12 IU/mL). Protein C enzymatic activity was measured with the peptide substrate H-D-Lys-Z-Pro-Arg-4-nitroanilide-diacetate (Chromozym PCa; 0.5 mM in 20 mM Tris, pH 7.4, 0.15 M NaCl, and 5 mg/mL BSA) at 37°C. The absorbance change at 405 nm was measured with a microplate reader (BioTeK, Seattle, WA).

### Real-Time Polymerase Chain Reaction (RT-PCR)

Total RNA was extracted from the pellet using a PureLink™ Micro-to-Midi Total RNA Purification System (Invitrogen™ by Life Technologies Corporation, Carlsbad, CA). Complementary DNA (cDNA) was synthesized from total RNA using Moloney Murine Leukemia Virus SuperScript II® Reverse Transcriptase (M-MLV RT, Invitrogen, Carlsbad, CA), and PCR analysis was performed using cDNA as the template in a reaction mixture (25 µL) containing a specific primer pair for each cDNA. The cDNA pool that was obtained using reverse transcriptase served as a template for subsequent PCR amplification. The amplification was performed on an ABI PRISM® 7900HT Sequence Detection System using a SYBR® PCR kit (both from Applied Biosystems, Foster, CA) according to the manufacturer’s protocols. The primer sequences used were as follows: TM: 5′-GGCCAAGATGGAGTACAAGTGC-3′ (forward) and 5′-CGCCAGTTAGCCATGGAATAGA-3′ (reverse); GAPDH: 5′-ATCCCTCCAAAATCAAGTGGG-3′ (forward) and 5′-TGAAGACGCCAGTGGACTCC-3′ (reverse). Real-time PCR analysis was performed with the following protocol: 1 cycle at 95°C for 10 min and 40 cycles of 95°C for 30 s, 60°C for 1 min, and 72°C for 1 min. GAPDH levels served as a control, and the ddCt algorithm was used to analyze relative changes in gene expression. A melt curve analysis was performed after amplification to verify amplicon accuracy.

### ROS Measurement

HaCaT cells were seeded in 96-well plates at a concentration of 1×10^5^ cells/mL and were preincubated with 10 µM N-acetyl-cysteine (NAC) for 2 h before UVB (3 mJ/cm^2^) exposure. The UV-irradiated cell cultures were treated with 10 µM 2,7-dichlorofluorescein diacetate (DCFH-DA; Sigma-Aldrich, St. Louis, MO) in PBS for 30 min. After incubation, the media was discarded, and the cells were washed with PBS. The fluorescence intensity was determined using a Spectramax M2e fluorescence plate reader (Molecular Devices, Silicon Valley, CA.) at 485 nm for excitation and 530 nm for emission. Relative fluorescence intensity was calculated using unexposed control cells as a standard.

### Promoter Magnetic Precipitation (PMP)

A biotinylated TM promoter (394 bp) was cloned into the pXP-1 vector by PCR-amplification using the forward primer 5′-CGTGCAGGCGCCGGGGAAAG-3′ and the reverse primer 5′-AGTCTCCGGTTCCCAGAGCTCTTGCAA-3′ with biotin conjugation on the 5′-end of the forward primer. The TM promoter fragment was conjugated with Dynabeads (Invitrogen, Carlsbad, CA,). The PMP assay was performed by incubating the immobilized TM promoter fragment with 200 g UVB-irradiated cell nuclear extracts. Samples were precleared with unconjugated Dynabeads in the presence of poly (dI:dC). After 3 h incubation at 4°C, the beads were collected by the magnetic apparatus and washed with the binding buffer containing 0.5 Nonidet P-40. Proteins that co-precipitated were eluted in SDS sample buffer and separated in SDS polyacrylamide gel electrophoresis. Subsequently, western blotting was performed to identify specific proteins in the magnetic precipitates.

### Chromatin Immunoprecipitation Assay

Chromatin immunoprecipitation analysis was performed using a commercial kit (Upstate, Lake Placid, NY) according to the manufacturer’s guidelines. Briefly, DNA and protein complexes were immunoprecipitated with primary antibodies that specifically recognized p53 or NF-κB. DNA was purified from the immuno-complex using a PCR purification kit (Qiagen, Carlsbad, CA). The purified DNA pellet was subjected to PCR, and the PCR products were resolved by 1.5% agarose gel electrophoresis and visualized by UV light. The following primer pairs that were specific to the TM promoter region (–394 to –58) were used: 5′-AGACCTTAGCGCGGTGTAGA-3′ and 5′-AGTAGCAGAGGAGCTCAGCG-3′.

### Western Blot Analysis

UVB-treated or untreated HaCaT cells were rinsed twice with cold PBS, and lysed in PBS containing 1% Nonidet P-40, 0.5% sodium deoxycholate, 0.1% sodium dodecyl sulfate (SDS), 5 µg/mL aprotinin, 100 µg/mL phenylmethylsulfonyl fluoride, 1 µg/mL pepstatin A, and 1 mM ethylenediaminetetraacetic acid at 4°C for 20 min. Total lysates were quantified using a microBCA kit (Thermo Fisher Scientific, Rockford, IL). Proteins (35 µg) were resolved by SDS-polyacrylamide gel electrophoresis and electrophoretically transferred to a PVDF membrane. The membrane was blocked in 5% fat-free milk in PBST buffer (PBS with 0.05% Tween-20) followed by incubation overnight with primary antibodies (mouse anti-human TM antibodies (1∶1000), mouse anti-human pERK antibodies (1∶1000), mouse anti-human ERK antibodies (1∶1000), mouse anti-human α-tubulin antibodies (1∶2000), mouse anti-human GAPDH antibodies (1∶2000) (Santa Cruz Biotech, CA, USA), mouse anti-human p53 antibodies (1∶1000), mouse anti-human NF-κB antibodies (1∶1000), mouse anti-human fibrillarin antibodies (1∶2000), and mouse anti-human lamin B1 antibody (1∶1000) (Abcam, Cambridge, MA) diluted in PBST buffer. The primary antibodies were removed, and the membrane was washed extensively in PBST buffer. Subsequent incubation with horseradish peroxidase-conjugated goat anti-mouse antibodies (1∶10000, Santa Cruz Biotech, Dallas, TX) proceeded at room temperature for 2 h. The membrane was washed extensively in PBST buffer to remove the secondary antibodies, and the blot was visualized with Enhanced ChemiLuminescence reagent (GE Healthcare Piscataway, NJ).

### Statistical Analysis

A negative control (sham-irradiated cells) was included in all of the experiments. The results were expressed as the mean ± SD of at least three experiments. Statistical analysis of the negative control data versus treatments and UVB treatment (*) or UVB treatment (#) was performed by Student’s *t* test. *p*<0.05 was considered to be statistically significant.

## Results

### Effects of UVB Irradiation on TM Expression in HaCaT Cells

**Figure 1:**
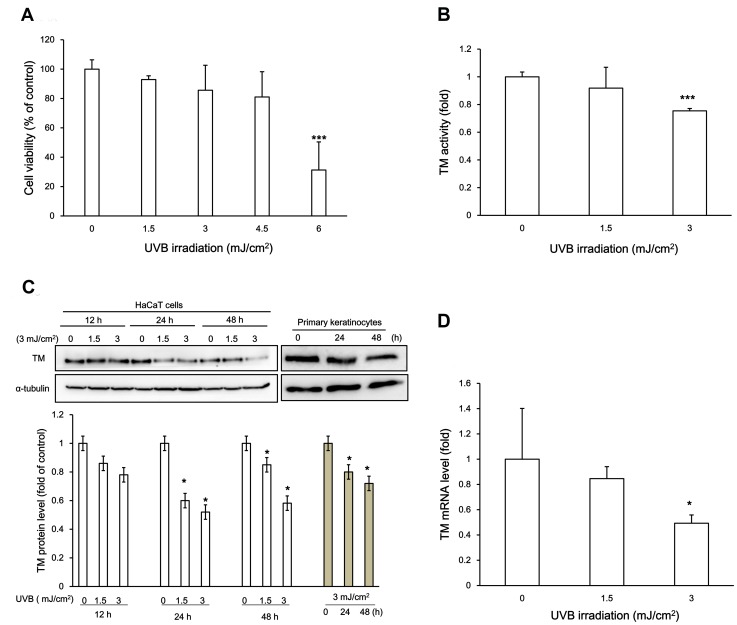
UVB irradiation decreased TM expression in HaCaT cells. (A) HaCaT Cells were exposed to UVB irradiation in a dose-response (0, 1.5, 3, 4.5, 6 mJ/cm^2^) Cell viability was examined by trypan blue exclusion. (B) HaCaT cells were exposed to 1.5 or 3 mJ/cm^2^ UVB for 24 h, and TM activity was determined by protein C activation analysis and expressed as fold change from the non-irradiated control. (C) (Upper panels) Western blot results of the UVB irradiation-mediated decrease in TM protein levels. α-tubulin served as an internal control. HaCaT cells were irradiated with 1.5, or 3 mJ/cm2 of UVB for 12, 24 or 48 h or without irradiation, respectively. On the other hand, primary human keratinocytes were irradiated with 3 mJ/cm2 of UVB for 24 or 48 h or without UVB treatment. (Lower panel) Quantification of Western blots from (C). The untreated cells at each time point served as a control for each time point. The results are presented as the mean ± standard deviation from three independent experiments. (D) Total RNA from UVB-irradiated HaCaT cells at the indicated doses was collected. TM mRNA levels were examined by real time RT-PCR using GAPDH as an internal control. (* *p*<0.05 *vs.* control; ***, *p*<0.001 *vs.* control; #, *p*<0.05 *vs.* 3 mJ/cm^2^ UVB-treated group).

To assess whether UVB irradiation resulted in keratinocyte cytotoxicity, we performed a UVB irradiation dose response in HaCaT cells and evaluated cell viability by trypan blue exclusion. As demonstrated in Fig. 1A, UVB irradiation doses in HaCaT cells ranging from 0 to 4.5 mJ/cm^2^ resulted in minor cytotoxicity, while 6 mJ/cm^2^ resulted in significantly decreased cell viability (*p*<0.05, t- test). The lower energy of UVB rays did not substantially induce a cell morphology change 24 h post-irradiation (data not shown). Thus, we applied 3 mJ/cm^2^ UVB irradiation for the following experiments.

To further elucidate the effect of UVB irradiation on TM expression, HaCaT cells were irradiated with UVB at different time points (12, 24 and 48 h post-irradiation), and the TM activity, mRNA and protein levels were determined. Decreased TM activity/expression (Fig. 1B and 1C) and mRNA levels (Fig. 1D) were first detected in the UVB irradiation range of 1.5–3 mJ/cm^2^. In addition, the expression of TM decreased after exposure to 3 mJ/cm2 of UVB for 24 h and 48 h in primary human keratinocytes (Fig. 1C). These results suggested that sub-lethal UVB irradiation dosage down regulates TM activity and protein levels, which may be because of downregulation of transcription regulation in HaCaT cells.

### UVB-induced TM Down-regulation was Mediated by ERK

**Figure 2:**
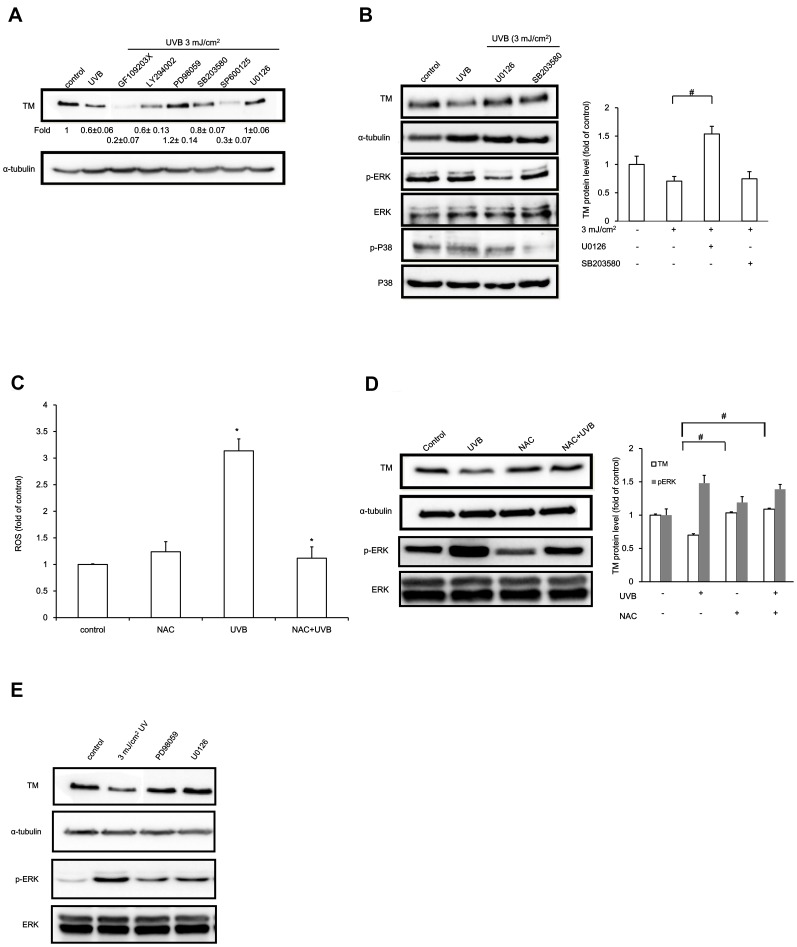
ERK activation mediates UVB-induced TM down-regulation in HaCaT cells. (A) HaCaT cells were pre-treated with 10 µM pharmacological inhibitors followed by UVB irradiation (3 mJ/cm^2^) for 24 h. TM protein levels were analyzed by Western blotting. α-tubulin served as an internal control. (B) HaCaT cells preincubated with 10 µM U0126 or SB203580 were irradiated with UVB (3 mJ/cm^2^). TM, pERK, and p-p38 protein levels were determined by Western blotting. α-tubulin, ERK, or p38 levels are used as loading controls, respectively. (C) Intracellular ROS levels were measured with 10 µM DCF-DA 30 min after UVB irradiation. The results are expressed as the mean ± S.D. of three independent experiments. (D) HaCaT cells were pretreated for 1 h with NAC followed by UVB irradiation (3 mJ/cm^2^) for 24 h, and TM and pERK protein levels were examined by Western blotting. α-tubulin and total ERK were used as loading controls. Relative TM or ERK phosphorylation levels were quantified and presented as the mean ± SD for three independent experiments performed in triplicate. #, *p*<0.05 compared with the 3 mJ/cm^2^ UVB-treated groups. (E) Human epidermal keratinocytes were pre-treated with 10 µM pharmacological inhibitors, UVB irradiated (3 mJ/cm^2^) and cultured for 24 h. TM and pERK protein levels were analyzed by Western blot. α-tubulin or ERK levels were used as loading controls, respectively.

UV irradiation results in ERK, JNK and p38 MAP kinase activation [25], [26]. Therefore, we investigated whether the UVB irradiation-induced TM down-regulation may be mediated by MAPK signaling. The HaCaT cells were pre-treated with inhibitors for 1 h followed by UVB irradiation (3 mJ/cm^2^) for 24 h in the presence of the inhibitors. As shown in the figure S1, UVB irradiation down-regulated TM protein levels. TM expression was not altered in response to suppression of ERK with ERK inhibitors PD98059, U0126, or PI3-kinase activation inhibitor LY294002. On the other hand, blocking of JNK activation with inhibitor SP600125 or abrogating protein kinase C activation with inhibitor GF 109203X inhibited the expression of TM. Moreover, our results revealed that treatment with a PI3K inhibitor (LY294002) or p38 inhibitor (SB203580) did not rescue UVB-induced TM protein level down-regulation. Treatment with a PKC inhibitor (GF109203X) or JNK inhibitor (SP600125) resulted in further TM protein level down-regulation after UVB irradiation. However, treatment with ERK inhibitors PD98059 or U0126 rescued the UVB-induced TM protein level down-regulation (Fig. 2A). UVB-induced ERK phosphorylation and TM suppression were further confirmed by Western blotting (Fig. 2B). These results suggested that UVB-induced TM down-regulation might be mediated by the ERK pathway.

Several reports have suggested that reactive oxygen species (ROS) accumulate following UV irradiation. We therefore evaluated ROS production in HaCaT cells after UVB irradiation. Intracellular ROS levels were significantly increased after UVB irradiation in HaCaT cells (Fig. 2C). Treatment with ROS scavenger NAC suppressed UVB-induced TM down-regulation (Fig. 2D). This result indicated that UVB-induced ROS production may decrease TM expression in HaCaT cells. To further elucidate whether the UVB irradiation-induced ROS production affected ERK signaling, we examined pERK levels. NAC pre-treatment attenuated UVB irradiation-induced ERK1/2 activation. Moreover, we found that UVB treatment decreased TM expression in primary human epidermal keratinocytes. Treating primary human epidermal keratinocytes with an ERK inhibitor prior to UVB exposure inhibited UVB-reduced TM production concomitant with UVB-induced phosphorylation of ERK1/2 (Fig. 2E). These results suggested that UVB-induced ROS might promote ERK activation and inhibit TM expression in keratinocytes.

### Involvement of p53 in UVB-induced TM Expression Down-regulation

**Figure 3:**
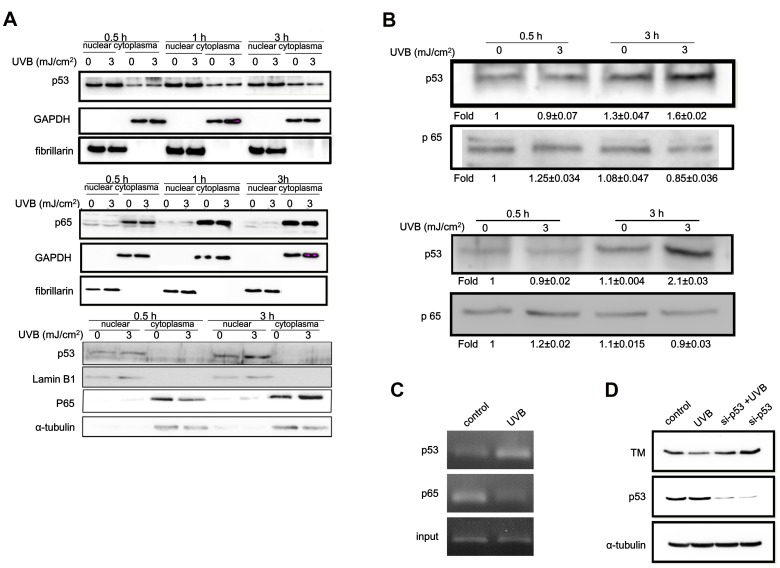
p53 mediated UVB-induced TM downregulation. (A) HaCaT cells (upper panel) or primary human epidermal keratinocytes (lower panel) were UVB irradiated (3 mJ/cm^2^) at the indicated time points, and cytosolic and nuclear fractions were harvested. p53 and p65 protein levels were determined by Western blotting. α-tubulin or lamin B1 levels were used as cytoplasmic or nuclear loading controls, respectively. (B) Nuclear extracts from UVB-irradiated HaCaT cells (left panel) or human epidermal keratinocytes (right panel) were incubated with proximal TM promoter DNA linked to Dynabeads. Protein-DNA complexes were allowed to form under native conditions and precipitated magnetically. Bound proteins were washed, eluted, and p53 and p65 levels were determined by Western blotting (C) HaCaT cells were UVB irradiated (3 mJ/cm^2^) for 3 h, and chromatin immunoprecipitation was performed. Chromatin was immunoprecipitated with anti-p53 or anti-p65 antibodies followed by amplification of TM promoter elements by PCR using specific primers. To verify equal loading, 1% of the precipitated chromatin was assayed as input. (D) HaCaT cells were transiently transfected with p53 siRNA or with a GAPDH short hairpin RNA (shRNA) as control. After transfection, cells were exposed to UVB (3 mJ/cm^2^) for 24 h, and cell lysate was harvested for Western blot analysis to assess p53 and TM levels. α-tubulin was used as a loading control.

UVB irradiation regulates the expression of a wide variety of genes that are mediated by transcription factor activation such as redox-sensitive transcription factor NF-κB or p53 [27], [28]. Therefore, we examined whether UVB irradiation activated these transcription factors and whether they were involved in UVB-induced TM down-regulation. We determined that UVB irradiation did not change NF-κB subunit p65 levels or subcellular localization (Fig. 3A). In contrast, elevated nuclear p53 protein levels appeared within 30 min after UVB treatment and lasted for at least 3 h in HaCaT cells. In primary human keratinocytes, the relative nuclear p53 amount was increased 3 h following UVB irradiation (Fig. 3A). However, though differences in normal cultured epidermal keratinocytes were observed, most of the p53 was detected primarily the cell nucleus but not in the cytoplasm compared with the HaCaT cells. In addition, cytosolic level of NF-κB p65 was increased and translocated from the cytosol to the nucleus 3 h after UVB treatment. (Fig. 3A).To further elucidate whether the increased nuclear p53 or p65 could bind to the TM promoter region and further suppress TM transcription, analysis of the 5′ flanking region of the human TM promoter sequence was performed with TFSEARCH software (http://molsun1.cbrc.aist.go.jp/research/db/TFSEARCH.html). These results revealed that putative NF-κB and p53 binding sites exist on the TM promoter. To examine the role of NF-κB and p53 in UVB-mediated TM expression in keratinocytes, the promoter region between −394 and +154 of TM was selected in the DNA-protein-binding assay. We determined that p53 but not NF-κB could physically interact with the TM promoter *in vitro* regardless of UVB irradiation status. UVB irradiation further increased p53 binding to the TM promoter in both types of keratinocytes (Fig. 3B). We further confirmed binding of p53 or NF-κB to the TM promoter region *in vivo* with a ChIP assay using HaCaT cell lysates with or without 3 mJ/cm^2^ UVB exposure. The ChIP assay revealed that p65 could bind to the TM promoter without UVB irradiation, while this binding was significantly decreased after UVB irradiation. However, UVB irradiation significantly increased p53 binding to the TM promoter sequence (Fig. 3C). These results confirmed *in vivo* binding of p53 to the TM promoter sequence upon UVB stimulation. To determine the physiological role of p53 in TM expression regulation, we utilized small interference RNA (siRNA) to perturb normal p53 function in HaCaT cells. The siRNA significantly decreased p53 protein levels and p53 knockdown rescued UVB irradiation-induced TM down-regulation (Fig. 3D). These results suggested that p53 acts as a transcriptional repressor in response to UVB irradiation, which decreases TM expression in HaCaT cells.

### UVB Regulates TM Expression via ERK Phosphorylation and p53 Nuclear Accumulation

**Figure 4:**
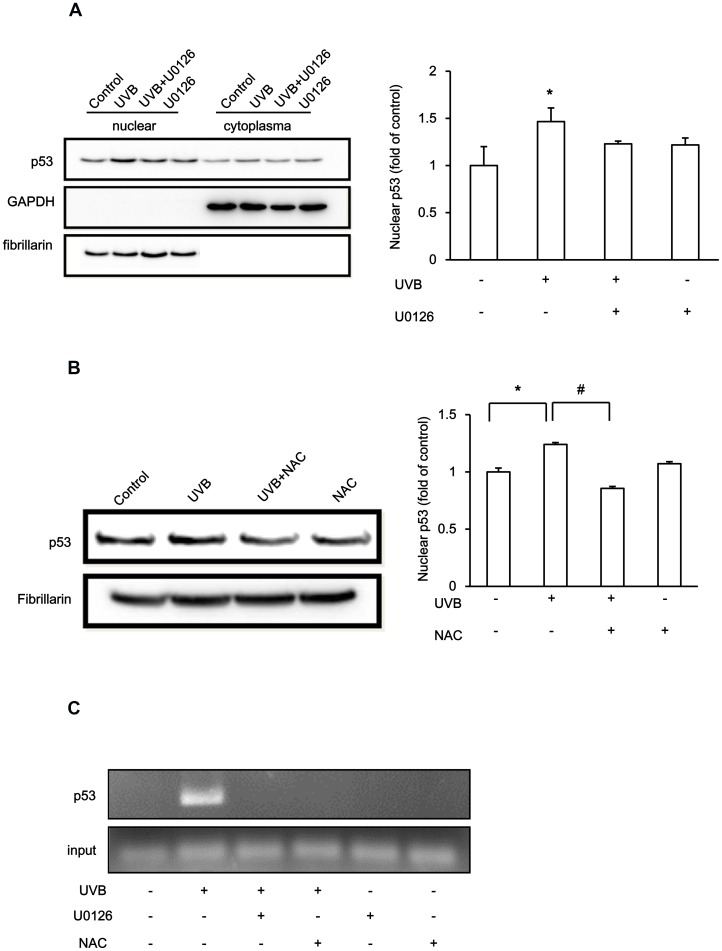
ROS-activated ERK Signaling Mediates p53-dependent TM regulation in UVB-treated HaCaT cells. (A) (Upper panels) HaCaT cells were treated with or without U0126 (10 µM) for 1 h followed by exposure to 3 mJ/cm^2^ UVB for 3 h. Cell lysate cytoplasmic and nuclear fractions were harvested separately p53 protein levels were analyzed. GAPDH and fibrillarin were used as cytoplasmic or nuclear loading controls, respectively. (Lower panel) Quantification of nuclear p53 levels from three independent experiments. The fold change of nuclear p53 is represented as the mean ± S.D. (B) (Upper panels) HaCaT cells were treated with or without NAC (10 µM) for 1 h and then exposed to 3 mJ/cm^2^ UVB for 3 h. The nuclear fraction was harvested and subjected to Western blotting for p53. Fibrillarin was used as an internal control. (Lower panel) Quantification of nuclear p53 levels from three independent experiments. The fold change of nuclear p53 is represented as the mean ± S.D. (*, *p*<0.05 *vs*. control, #, *p*<0.05 vs. 3 mJ/cm^2^ UVB-treated group). (C) HaCaT cells were pretreated with U0126 or NAC (10 µM) followed by UVB treatment (3 mJ/cm^2^) for 3 h. Chromatin immunoprecipitation was performed. Chromatin was immunoprecipitated with anti-p53 antibodies and the TM promoter region was amplified by PCR using specific primers. To verify equal loading, 1% of the precipitated chromatin was assessed as input.

**Figure 5:**
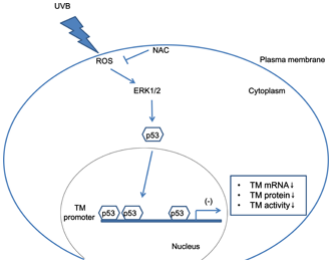
The proposed model of UVB-mediated TM suppression. UVB-induced ROS accumulation and ERK activation lead to nuclear p53 accumulation, which binds to the TM promoter and inhibits TM expression in HaCaT cells.

To further investigate the mechanism of UVB irradiation-induced p53 nuclear up-regulation and subsequent binding to the TM promoter to suppress TM expression, we examined whether ERK activation and ROS might be involved in p53 nuclear localization after UVB irradiation. UVB irradiation increased p53 nuclear accumulation, which was blocked by U0126 treatment (Fig. 4A), suggesting the involvement of ERK signaling. In addition, UVB irradiation-induced nuclear p53 accumulation could be abolished by the pre-treatment with ROS scavenger NAC (Fig. 4B), suggesting a role for ROS in the regulation of UVB-induced nuclear p53 accumulation. The ChIP analysis was further performed to confirm this phenomenon. The results demonstrated that UVB irradiation-induced p53 binding to the TM promoter, which was inhibited by ERK inhibitor U0126 or ROS scavenger NAC pre-treatment (Fig. 4C). Thus, those results revealed that the UVB*-*induced intracellular ROS accumulation, which subsequently activated ERK signaling and facilitated p53 binding to the TM promoter, thus suppressing keratinocyte TM expression (Fig. 5).

## Discussion

Human epidermis is routinely subjected to photo-damage, which is usually induced by UV irradiation. The principal pathogenic mechanism of UVB is oxidative stress accumulation and ROS generation, which can directly damage cell membranes and proteins [29], [30]. Antioxidants block certain pathways with proven beneficial effects on UV-induced skin aging [31]. Here, we report that UVB downregulates surface TM in cultured human keratinocytes, which is mediated by transcriptional regulation. TM expression is restored after antioxidant NAC treatment (Fig. 1). Therefore, our finding provides significant insight into TM downregulation by UVB exposure, a mechanism that may be implicated in photo-aging. Although using a relatively low dose (3 mJ/cm^2^) for which a transition point between no observable effect and a weak toxic response would contribute to statistical variability, the reduced trend of UVB-induced TM downregulation at the mRNA and protein level was consistent between the two types of keratinocytes that were assessed.

UV irradiation causes human skin photo-aging by downregulating a number of intracellular and extracellular proteins via multiple cell signaling pathways [32], [33]. Environmental stress-induced signaling cascades activate mitogen-activated protein kinase cascades such as ERK, c-Jun N-terminal kinase (JNK), and p38 MAPK [26], [34]. We demonstrated here that TM is downregulated by sub-cytotoxic UVB irradiation and that the TM promoter is negatively regulated by ERK-dependent signaling pathways. p53 binding to the TM promoter was observed, which is likely responsible for reducing TM expression. In 2008, Iwata *et al.* demonstrated that UVB induced TM expression via the p38 signaling pathway in HaCaT keratinocytes. In their study, TM expression was monitored in serum starved HaCaT cells, and the UVB dosage they used was higher (10 mJ/cm^2^) than the dose in our study [35]. Indeed, 10 mJ/cm^2^ UVB induced apoptosis and ultimately cell death [36]–[38]. Thus, further studies on investigating the regulatory mechanism of TM expression in response to environmental UVB using normal human keratinocytes and skin tissues are needed to determine their biological relevance.

UV irradiation stimulates signaling pathways that activate transcription factors including AP-1, NF-κB, and p53 [39], [40]. In the present study, we demonstrated that activated ERK is involved in UVB-induced inhibition of TM expression in keratinocytes. Specifically, the transcription factor p53 mediated this effect. Transfection of p53 siRNA reduced the UVB-mediated suppression of TM gene transcription. Although previous studies demonstrated that NF-κB inhibited TM expression by competing with the limited pool of nuclear p300 in endothelial cells [41], [42]. In addition, several putative NF-κB binding sites were found in the TM promoter regions. Conversely, NF-κB binding to the TM promoter was decreased by UVB irradiation. We speculated that low-dose UVB exposure reduced chromosomal-bound NF-κB, which would modulate TM expression [43].

The expression pattern of TM in skin has been reported during keratinocyte differentiation [44]. However, the role of p53 in TM transcription suppression at the molecular level remains to be elucidated. Our study suggested that p53 might play a role as a transcriptional repressor to down-regulate TM expression by repressing distinct TM promoter regions. It has been demonstrated that p53 suppresses TM expression by transcriptional repression of Kruppel-Like Factor 2 (KLF-2) in endothelial cells [45]. KLF-2 is a major transcription factor that regulates TM expression and activity [46]. Alternatively, p53 may recruit transcriptional repressors to the TM promoter to inhibit its activity. In 2010, Kao *et al*. demonstrated that transcription factor Snail can directly inhibit TM expression in HaCaT cells, which results in epithelial-mesenchymal transition and tumorigenesis [47]. It is possible that p53-mediated TM suppression via Snail recruitment to the TM promoter in response to UVB irradiation [48]. In addition, p53 may bind to its target genes and recruit histone deacetylases, which subsequently deacetylate histone H3 and H4 and decrease gene expression [49]–[51]. This hypothesis is supported by Rong et al., who demonstrated that HDAC4 serves as a co-repressor to regulate TM expression in endothelial cells [52]. This suppressive capacity is exerted through direct sequence-specific binding of activated p53 to *bona fide* target sites, which sterically inhibits TM transcriptional activation. The current study demonstrates that ERK and p53 operate together in UVB–induced TM transcriptional regulation in normal keratinocytes. Our findings may suggest a potential function of TM in photo-damage-induced skin disorders.

In summary, UVB irradiation decreased active TM protein levels in human keratinocytes. The UVB irradiation-induced TM down-regulation was mediated by reactive oxygen species-mediated ERK activation and, subsequently, p53-mediated transcriptional repression via p53 binding to the TM promoter (Fig. 5).

## Supporting Information

Figure S1 Effects of inhibitors on TM protein expression in HaCaT cells. The cells were treated with inhibitors (10 µM) for 1 h then washed with PBS and were mock-irradiated. The cells were cultured in inhibitor containing medium and harvested at 24 h. The cell lysates were prepared and western analysis was performed with a TM antibody. Equal loading was monitored by re-probing the membrane with an anti α-tubulin antibody.
(TIF)
