# Supplementary figures and images for: UVB Irradiation Regulates ERK1/2- and p53-Dependent Thrombomodulin Expression in Human Keratinocytes
Source: PLoS One. 2013 Jul 2;8(7):e67632. doi: 10.1371/journal.pone.0067632 (PMC3699658; doi:10.1371/journal.pone.0067632)

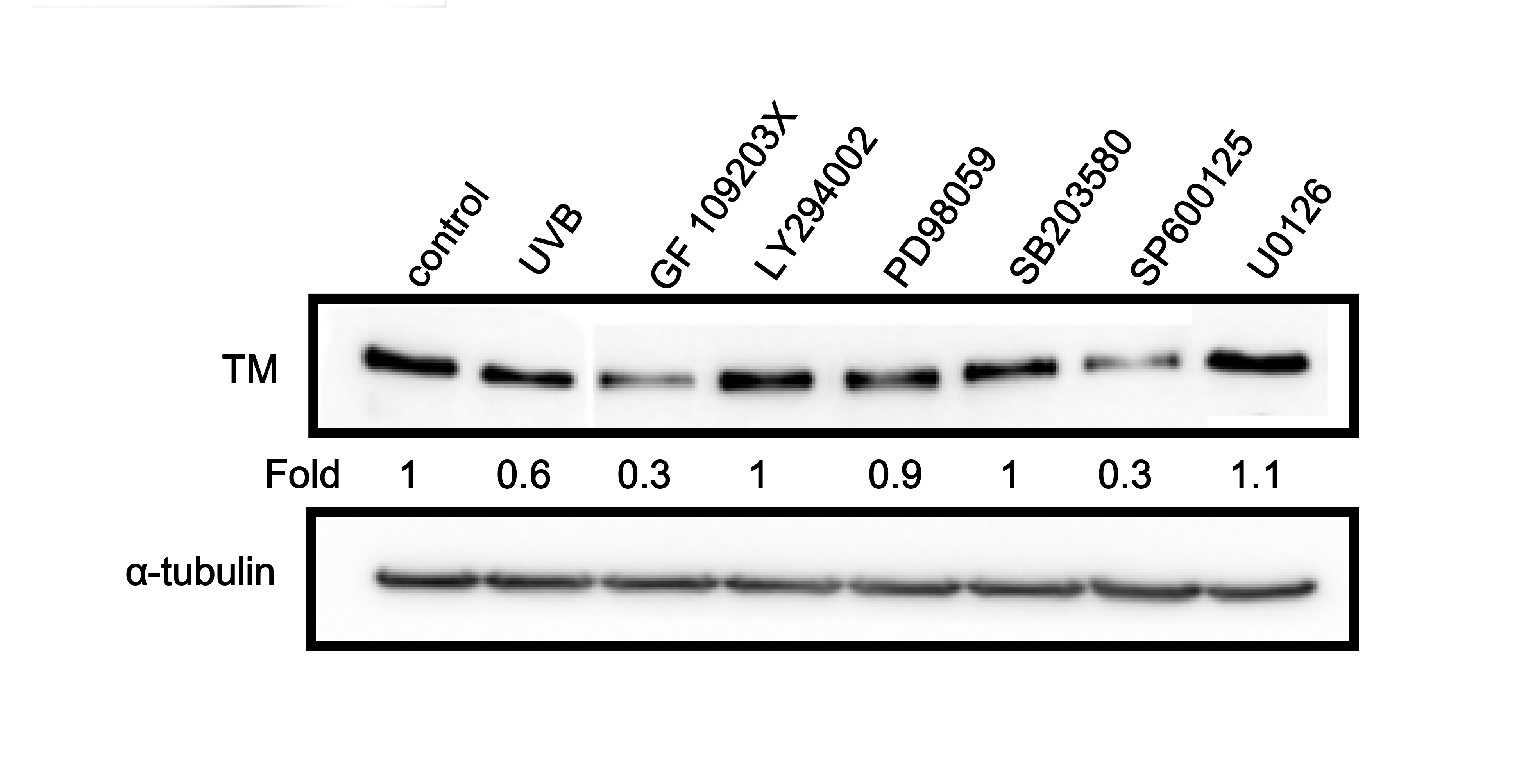

Supplement: Figure S1 — Effects of inhibitors on TM protein expression in HaCaT cells. The cells were treated with inhibitors (10 µM) for 1 h then washed with PBS and were mock-irradiated. The cells were cultured in inhibitor containing medium and harvested at 24 h. The cell lysates were prepared and western analysis was performed with a TM antibody. Equal loading was monitored by re-probing the membrane with an anti α-tubulin antibody. (TIF) [file pone.0067632.s001.tif]
